# IMRT in the treatment of locally advanced or inoperable NSCLC in the pre-durvalumab era: clinical outcomes and pattern of relapses, experience from the Oscar Lambret Center

**DOI:** 10.3389/fonc.2023.1236361

**Published:** 2023-09-20

**Authors:** Thomas Le Roy, Jennifer Wallet, Maël Barthoulot, Clémence Leguillette, Thomas Lacornerie, David Pasquier, Eric Lartigau, Florence Le Tinier

**Affiliations:** ^1^ Academic Department of Radiation Oncology, Centre Oscar Lambret, Lille, France; ^2^ Department of Biostatistics, Centre Oscar Lambret, Lille, France; ^3^ Department of Medical Physics, Centre Oscar Lambret, Lille, France; ^4^ CRIStAL UMR CNRS 9189, Lille University, Lille, France

**Keywords:** IMRT, locally advanced or inoperable NSCLC, NSCLC, radiochemotherapy, radiotherapy, relapses, isolated nodal failure

## Abstract

**Background:**

Intensity-modulated conformal radiotherapy (IMRT) has become the technique of choice for the treatment of locally advanced or inoperable non-small cell lung cancer (NSCLC). Nevertheless, this technique presents dosimetric uncertainties, particularly in treating moving targets such as pulmonary neoplasms. Moreover, it theoretically increases the risk of isolated nodal failure (INF) due to reduced incidental irradiation.

**Objective:**

The objective of this study was to evaluate the efficacy and safety of IMRT in patients with inoperable NSCLC and to describe the pattern of relapses.

**Methods:**

Patients with locally advanced NSCLC treated with radiotherapy and chemotherapy between 2015 and 2018 at the Oscar Lambret Center were retrospectively included in the study. Overall and progression-free survival were estimated using the Kaplan–Meier method. The cumulative incidence of the different components of relapse was estimated using the Kalbfleisch and Prentice method. Prognostic factors for relapse/death were investigated using the Cox model. A comparison with literature data was performed using a one-sample log-rank test.

**Results:**

Seventy patients were included, and 65 patients (93%) had stage III disease. All the patients received chemotherapy, most frequently with cisplatin and navelbine. The dose received was 66 Gy administered in 33 fractions. The median follow-up and survival were 49.1 and 39.1 months, respectively. A total of 35 deaths and 43 relapses, including 29 with metastatic components, were reported. The overall survival rates at 1 and 2 years were 80.2% (95% confidence interval 68.3%-88.0%) and 67.2% (95% confidence interval 54.2%-77.3%), respectively. Locoregional relapse was observed in 14 patients, including two INF, one of which was located in the lymph node area adjacent to the clinical target volume. Median relapse-free survival was 15.2 months. No variable was statistically associated with the risk of relapse/death in multivariate analysis. Seven patients (10%) experienced grade 3 or higher toxicity.

**Conclusion:**

The use of IMRT for locally advanced or inoperable NSCLC led to favorable long-term clinical outcomes. The rate of locoregional relapse, particularly isolated lymph node failure, was low and comparable with that of the three-dimensional radiotherapy series, as was the rate of early and late toxicities.

## 1 Introduction

Lung cancer is the leading cause of cancer mortality worldwide (1). The therapeutic arsenal for lung cancer has expanded in recent years, with improvement in overall survival (OS), but its management is a public health concern. Radiotherapy is a major therapeutic modality for the treatment of locally advanced or inoperable non-small cell lung cancer (NSCLC) (representing 85% of lung cancers), for which concomitant radiochemotherapy is recommended (2–4). Since the introduction of intensity-modulated conformal radiotherapy (IMRT), it has become a standard treatment for several indications (5–10). Despite the lack of evidence of its superiority over three-dimensional conformal radiotherapy (3D-CRT) in randomized controlled trials, IMRT is widely used worldwide for the treatment of lung cancers because of its better target volume coverage, which is associated with a decrease in dose delivered to the organs at risk (11–14).

Several retrospective studies have compared 3D-CRT with IMRT for the treatment of NSCLC and found conflicting results in terms of efficacy and toxicity (15–25). The only prospective cohort study (23) was a secondary analysis of RTOG 0617. This study reported a lower toxicity rate in the IMRT group with similar clinical outcomes, despite the presence of poor prognostic factors, such as larger tumor size or more advanced than stage IIIB.

For several years, prophylactic mediastinal irradiation, also called “elective nodal irradiation,” has been abandoned in favor of the treatment of invaded lymph nodes only, also called “selective irradiation” or “involved field radiation therapy.” This paradigm change followed the publication of selective irradiation trials that described similar relapse rates with reduced toxicities (26–30). Notably, these trials were all performed using 3D-CRT. Selective irradiation allows a part of prophylactic mediastinal irradiation called “incidental irradiation,” which could theoretically eliminate micro-metastases from the tumor environment.

With the advent of IMRT, which is much more conformal and allows for larger dose gradients, “incidental irradiation” is reduced, and a theoretical increase in the risk of isolated nodal failure (INF), which is a mediastinal lymph node relapse outside the clinical target volume (CTV) without relapse in the treatment field, is expected. In addition to this uncertainty in the treatment of lymph node disease, IMRT also provides dosimetric uncertainty for the treatment of primary tumors because of its mobility during breathing. These dosimetric uncertainties are more important than in 3D-CRT (31, 32). This leads to a greater theoretical risk of underdosing and, therefore, relapse of the primary tumor, especially in the absence of respiratory motion management. We describe a large retrospective series of patients treated with radiochemotherapy with IMRT for locally advanced or inoperable NSCLC between 2015 and 2018. This study aimed to evaluate the efficacy and safety of IMRT and to describe the pattern of relapse.

## 2 Materials and methods

### 2.1 Study population

All consecutive patients who met the eligibility criteria were retrospectively included in this study. The inclusion criteria were as follows: patients treated with IMRT for locally advanced or inoperable NSCLC from January 1, 2015 to December 31, 2018 in the radiotherapy department of the Oscar Lambret Center in Lille, patients aged ≥18 years, a systematic extension workup including positron emission tomography–computed tomography (PET-CT) scan and brain imaging, curative IMRT treatment with a planned dose of at least 60 Gy, and associated chemotherapy. Inclusion was voluntarily discontinued in December 2018 due to the authorization of durvalumab in consolidation from February 2019 to maintain a homogeneous population. The exclusion criteria were exclusive radiotherapy, 3D-CRT treatment, and synchronous second primary and metastatic diseases on extension. Patients were required to provide consent to the use of their medical data.

### 2.2 Treatment and follow-up

All patients underwent PET-CT and brain imaging as part of the extension workup. Additional endobronchial ultrasound was performed in cases of suspicious lymph node fixation on PET. Conventional centering scans with 2.5-mm slice thicknesses were performed without 4D-CT acquisition. PET-CT contributed to the tumor and lymph node GTV contouring phases. Selective lymph node irradiation was performed, targeting only PET- and/or endobronchial ultrasound-positive mediastinal areas and nodes larger than 1 cm on the simulation scan. A margin of 6-8 mm was added around the tumor GTV depending on the histology [CTV = (tumor GTV + 6-8 mm) + affected lymph nodes]. A 5-mm margin was added to the CTV to define the PTV.

Treatments were performed using a rotational IMRT technique on helical tomotherapy machines (Accuray Tomotherapy^®^) without tumor motion management. The prescribed dose was 60-66 Gy in 2 Gy fractions, concomitantly with chemotherapy if possible and sequentially if not. The dose constraints were as follows: V20 lungs, <30%; V30 lungs, <20%; V50 esophagus, <30%; and Dmax spinal cord, <45 Gy.

Radiation therapy was prescribed at 95% isodose and verified using the D95, representing the minimum dose received by 95% of the PTV. Homogeneity was also evaluated using D98 and D2%, representing the minimum dose received by 98% of the PTV in Gy and the maximum dose received by at least 2% of the PTV in Gy, respectively.

Follow-ups were performed every 3 months initially, then every 6 months, alternating with our oncopneumologist colleagues, or more regularly in cases of unusual treatment toxicity. Reassessments were performed using PET-CT or CT-scan, with brain imaging in case of a call point.

### 2.3 Data collection and regulatory aspects

Data were retrospectively collected from the medical records. The initial disease stage was defined according to the Union for International Cancer Control TMN 7th version. Follow-up radiology and nuclear medicine images were used to identify relapses. Relapses were classified into tumor, lymph node in the treatment field, lymph node out of the field, and metastasis. In cases of isolated local or locoregional relapse, the images were compared with the radiotherapy treatment plans. Out-of-field node relapses were considered as such in cases of localization entirely outside the CTV and were divided into two categories (adjacent and non-adjacent) by using the thoracic anatomy atlas of Chapet et al. (33) INF was specifically studied. Due to the difficulty in distinguishing a single metastasis from a second lung cancer, relapse as a single nodule was classified as metastatic relapse. Toxicities were recorded during treatment and at each follow-up visit and defined according to the Common Terminology Criteria for Adverse Events (CTCAE) v5.0.

The study complies with the “reference methodology” MR004 adopted by the French Data Protection Authority. The patients granted consent to the use of their clinical data for research purposes. The study was approved by an international review board (CEC-2022-004). No funding was received for the study.

### 2.4 Endpoints

The primary objective of this study was to describe OS. OS was defined as the time from IMRT to death from any cause, and data of patients who were known to be alive on the date of last follow-up were censored.

The secondary objectives were to describe the topography of relapses, describe progression-free survival (PFS), identify prognostic factors of PFS, describe the toxicities of IMRT, and compare our data with those of similar series. PFS after RT was defined as the time from the start of IMRT to the date of progression (local or distant) or death from any cause. Data of patients who were still alive and had not progressed were censored at the date of the last follow-up visit.

### 2.5 Statistical analysis

The median follow-up period was estimated using the inverse Kaplan–Meier method (Schemper) from the start of IMRT to the date of the last follow-up. OS and PFS were estimated using the Kaplan–Meier method (34). The cumulative incidence of each relapse component (local, lymph node, distant relapse, etc.) was estimated using the Kalbfleisch–Prentice method (35), which considers prior events as competing events.

Potential prognostic factors for PFS were evaluated using univariate Cox models to estimate hazard ratios. A multivariable Cox model was then performed using a selection step to select variables associated with a p-value <0.2 in the univariate analyses. In the final multivariate model, all tests were performed with a two-sided alpha level of 0.05.

We compared our OS data with those of a subgroup of patients treated without dose escalation in the RTOG 0617 study by Bradley et al. (36). To obtain comparable data, only patients with stage IIIA or IIIB disease were included in the analysis. We estimated the coordinates of the survival curve using the Datathief tool (37) and then generated individual survival data. The one-sample log-rank test was used to compare the two curves. We first approximated the data extracted from Bradley et al. using a parametric distribution according to a log-normal distribution. We then calculated the cumulative hazard function and applied it to the data.

Estimates were provided with their 95% confidence intervals (95% CI). Analyses were performed using STATA software (version 17.0; StataCorp. LLC, College Station, TX, USA).

## 3 Results

### 3.1 Clinical and irradiation population characteristics

Seventy patients who underwent IMRT for locally advanced or inoperable NSCLC between January 2015 and December 2018 were included. Population characteristics are presented in **Table 1**. The median age at diagnosis was 62 years (range, 39-83); 83% were men and 97% were smokers, 46% of whom were active smokers. The majority of patients (65 patients, 93%) had locally advanced stage III tumors (28 stage IIIA, 28 stage IIIB, and 9 stage IIIC), and five patients (7%) with stage II disease were also included in the analysis. These patients were considered inoperable due to their comorbidities and required concurrent radio-chemotherapy. The median dose delivered was 66 Gy in 33 fractions (range, 56-66), with a median PTV size of 370.5 cc (range, 76.8-921.8). In 71% of cases (50 pts), chemotherapy was performed concomitantly. The most commonly used chemotherapy doublet was the combination of cisplatin and vinorelbine (36 patients, 51%). Six patients received adjuvant durvalumab under temporary authorization for use, and only one of them relapsed and died of lung cancer.

**Table 1:** Population characteristics (N=70).

Characteristic (N=70)	n (%)
Age (median, range)	62 (39-83)
Sex	
Men	58 (83)
Women	12 (17)
Smoker (MD=1)	
Active	32 (46)
Weaned	35 (51)
No	2 (3)
COPD	
No	45 (64)
Yes	25 (36)
Stage 1	5 (7)
Stage 2	9 (13)
Stage 3	6 (9)
Stage 4	2 (3)
MD	3 (4)
Stage	
II	5 (7)
IIA	1 (1)
IIB	4 (6)
III	65 (93)
IIIA	28 (40)
IIIB	28 (40)
IIIC	9 (13)
Histology	
Adenocarcinoma	33 (47)
Squamous cell carcinoma	32 (46)
Sarcomatoid	1 (1)
Large cells	1 (1)
NSCLC unspecified	3 (4)
Lobe	
Inferior	14 (22)
Median	2 (3)
Superior	48 (75)
Chemotherapy	
Concomitant	50 (71)
Sequential	20 (29)
Delivered dose (Gy) (median, range)	66 (56-66)
PTV size (median, range)	370.5 (76.8-921.8)
MD: missing data	

The median of D95 PTV, D98 PTV and D2% were 63.2 (44.3-65.5), 61.9 (22.3-65.1), and 68.4 (62.0-71.0), respectively. The median V5 and V20 lung were 58.0 (9.2-99.6) and 23.0 (5.0-63.2), respectively. The median esophageal V50 esophagus was 21.2 (0.0-68.3).

### 3.2 Oncologic outcomes

**Figure 1:**
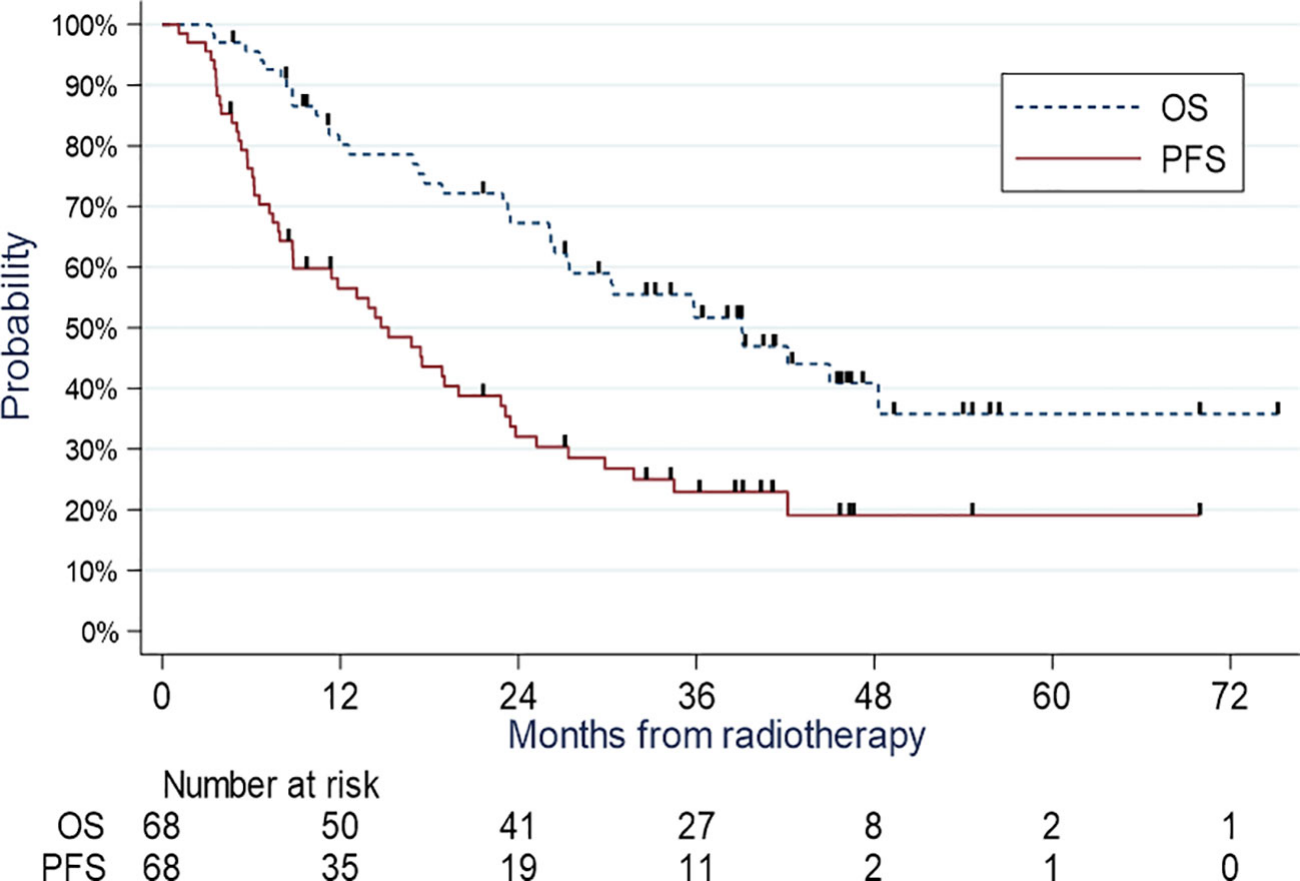
OS and PFS.

Of the 70 patients included, two were excluded from the survival analysis because they were lost to follow-up immediately after treatment. The median follow-up was 49.1 months (range, 45.5-56.2). Overall, 35 deaths were reported. Of these, 22 were disease-related, three were unrelated, and 10 were of unknown etiology. The median OS was 39.1 months (95% CI, 26.4-not achieved). The OS at 12 months was 80.2% (95% CI, 68.3-88.0) and 67.2% at 24 months (95% CI, 54.2-77.3) (**Figure 1**).

Overall, 43 relapses, including 14 local and/or regional, 18 metastatic, and 11 local, regional, and metastatic relapses, were reported. Thus, we observed 29 relapses of the metastatic components. In total, 50 patients relapsed or died, of whom 7 died without prior relapse. The median PFS was 15.2 months (95% CI, 36.9-50.6). The PFS at 12 months was 56.5% (95% CI, 43.8-67.4), 32.0% at 24 months (95% CI, 20.9-43.6), and 19.1% at 5 years (95% CI, 9.4-31.3) (**Figure 1**). In univariate Cox models, we found a significant association between PFS and lymph node involvement (N0-1 vs N2-3, p=0.046), presence of cardiovascular comorbidities (p=0.02), tumor size (p=0.18), PTV (p=0.04), and D98 PTV (p=0.10). In multivariate Cox models, none of these factors were significantly associated with PFS. However, lymph node involvement (N0-N1 vs N2-N3) was close to significance with a hazard ratio of 2.46 (0.97-6.25, p=0.059).

**Figure 2:**
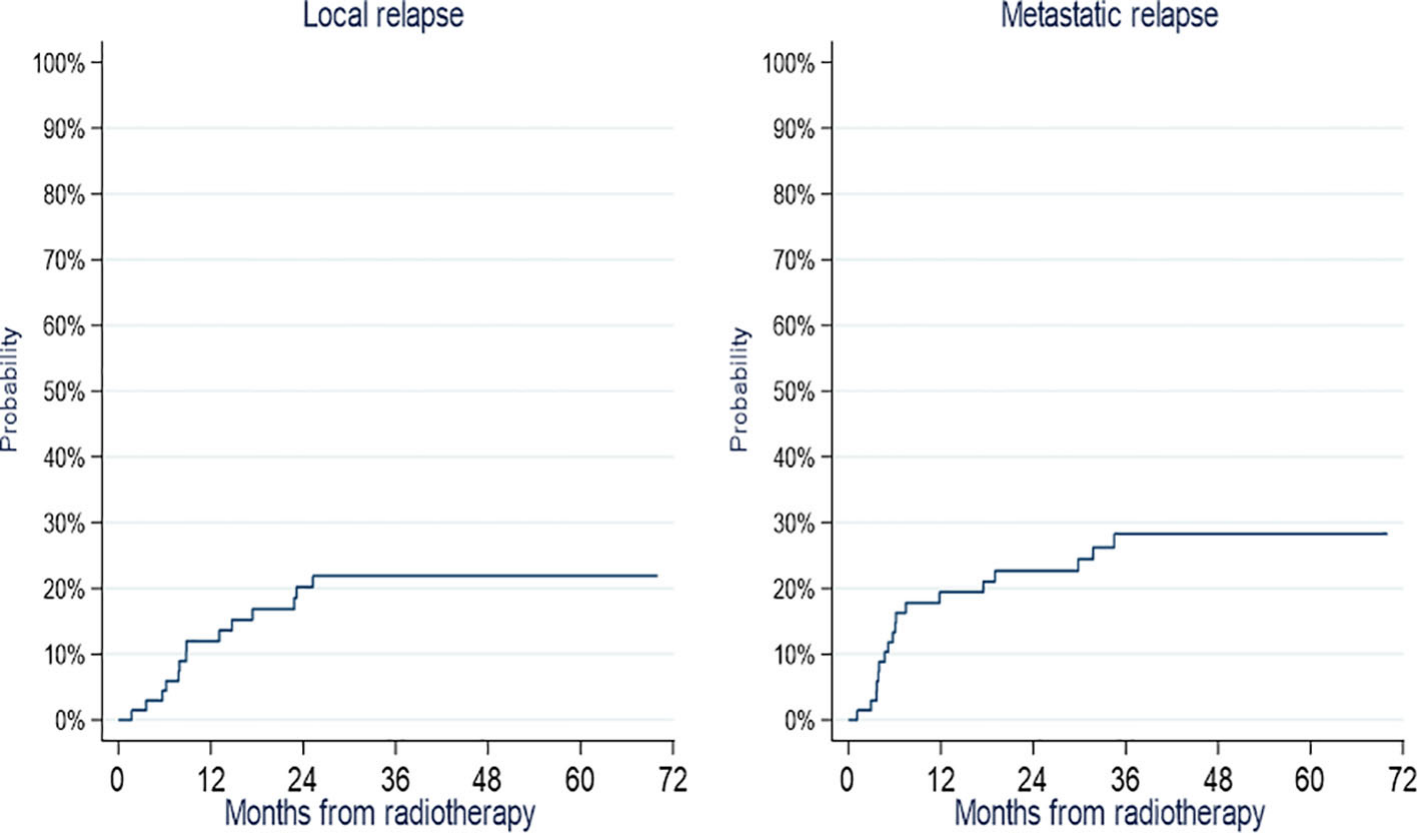
Cumulative incidence of local and metastatic relapses.

The cumulative incidence of local and metastatic relapses at 5 years were 21.9% (95% CI 12.7-32.7) and 28.3% (95% CI 17.8-39.7), respectively (**Figure 2**).

### 3.3 Relapse patterns

Details of relapse are presented in **Table 2**. Two patients presented with INF. Both relapses occurred in a single node outside the field, of which only one was adjacent to the treatment field. This adjacent INF was a relapse in the 11 L area, and 4 L, 5 L, and 10 L areas were initially invaded and included in the PTV. Two patients presented with abdominal lymph node relapse and were therefore considered metastatic. Seven patients presented with an isolated tumor relapse, of which two in the lower lobe and four had mixed tumor and lymph node relapses in the field. Eleven patients presented with relapse in the field of irradiation (tumor and/or mediastinal lymph nodes) without distant relapse.

**Table 2:** Relapse details (N=43).

Type of relapse	n (%)
Isolated primary tumor	7 (16)
Upper lobe	5 (12)
Lower lobe	2 (5)
Isolated nodal failure	2 (5)
In field	0
Out of field (INF)	2 (5)
Adjacent	1 (2.5)
Non-adjacent	1 (2.5)
Locoregional (tumor and nodal relapse)	5 (12)
In field	4 (9.5)
Out of field	1 (2.5)
Distant metastases	18 (42)
Locoregional with distant metastases	11 (26)
Total relapses	43 (100%)

### 3.4 Toxicity

The toxicities reported are shown in the **Table 3**. Overall, seven (10%) patients presented with grade 3 toxicity. We counted 1 radiation esophagitis requiring hospitalization and enteral nutritional support, 5 radiation pneumonitis requiring oxygen therapy, antibiotics, and corticosteroids, and 1 radial esophageal stricture requiring multiple endoscopic dilations. None of the patients experienced grade 4 or 5 toxicity. One patient presented with grade 2 plexitis, necessitating anti-inflammatory treatment. Two patients presented with esobronchial fistulas requiring a covered stent. Eight (11%) patients had no radiation-related toxicity at all. No acute or late cardiac toxicities, such as coronary events, have been reported.

**Table 3:** Early and late toxicities reported.

Toxicities reported	n (%)
Early toxicities	
Radiation pneumonitis	35 (50)
Grade 1	29 (41)
Grade 2	1 (1)
Grade 3	5 (7)
Grade 4-5	0
Radiation esophagitis	43 (61)
Grade 1	26 (37)
Grade 2	16 (23)
Grade 3	1 (1)
Grade 4-5	0
Pericarditis	0
Late toxicities	
Esophageal stricture	4 (6)
Grade 1	0
Grade 2	3 (4)
Grade 3	1 (1)
Radiation plexopathy	1 (1)
Grade 1	0
Grade 2	1 (1)
Oesobronchial fistula	2 (3)
Cardiovascular event	0

### 3.5 Comparison to literature data

**Figure 3:**
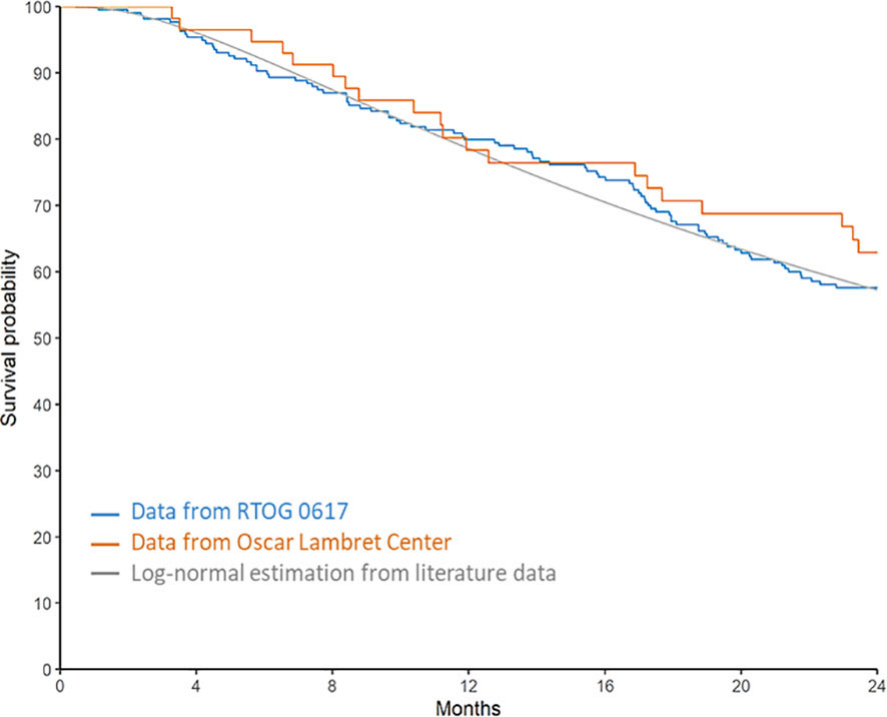
Comparison of overall survival with the RTOG 0617 study.

For this analysis, 57 patients with stage IIIA or IIIB disease were included and compared with patients from the 60 Gy subgroup of the RTOG 0617 study. The two OS curves are shown in **Figure 3**. The p value of the one-sample log-rank test was 0.94. Therefore, we cannot conclude that the survival observed in our study was better than that in the 60 Gy subgroup of the RTOG 0617 study.

## 4 Discussion

The median OS was 39.1 months; the 2-year OS and PFS rates were 67.2% (95% CI 54.2-77.3) and 32% (95% CI 20.9-43.6), respectively, with acceptable grade III toxicities (10%).

Our efficacy results are higher than those obtained by previous studies. In the 60 Gy subgroup of the RTOG 0617 study (36, 38), the median survival was 28.7 months and the 2-year PFS was 25.2% (95% CI 19.7-31.1), with treatments performed either in 3D-CRT or IMRT. In the secondary analysis of this study by Chun et al. (23), the subgroup of patients treated with IMRT showed a 2-year overall survival rate of 53.2% (95% CI 46.4-59.6). Similarly, in the control arm of the PACIFIC trial (39) with patients treated for locally advanced NSCLC with radiochemotherapy, without adjuvant durvalumab, the median survival was 29.1 months and the 2-year survival and PFS rates were 55.6% (95% CI 48.9-61.8) and 25.1% (95% CI 19.3-31.2), respectively. Nevertheless, our study and population present certain differences from those of the two previous studies. First, we selected patients by performing a systematic PET scan and brain imaging during the extension workup (23, 36, 38). In addition, systemic treatments for metastatic tumors have improved over the years, particularly targeted therapies and immunotherapies. Notably, our patients were treated between January 2015 and December 2018, nearly 8 years after the RTOG 0617 cohort (2007–2011), during which treatment strategies have evolved. It is also important to note that the 5 stage II patients included in the study did not artificially improve overall survival, as 3 of them died of their lung cancer.

However, relapse-free survival may have been artificially enhanced by the retrospective, real-world nature of this study, with examinations that may have been delayed, and relapses detected later than those in the clinical research protocol. In addition, the median survival may have been artificially overestimated because of the large amount of censored data after 2 years of follow-up, related to late inclusion in the study. This difference may not allow our median survival rate to be easily compared with that reported in literature.

Most relapses in locally advanced or inoperable NSCLC are metastatic, which was also confirmed in our series, with 29 of 43 relapses having a metastatic component. The cumulative incidence of metastatic relapse at 2 and 5 years were 22.7% (95% CI 13.5-33.3) and 28.3% (95% CI 17.8-39.7), respectively. Two of the 70 patients (2.9%) presented with INF. These two relapses were mediastinal, outside the treatment field, one of which was adjacent to the lymph nodes included in the CTV. Comparable rates were found in the selective 3D-CRT irradiation studies by De Ruysscher et al. (26) and Belderbos et al. (27), with 2% and 2.3%, respectively. Our results are also comparable with those of the selective IMRT irradiation study by Martinussen et al. (40), which found four INF out of 183 patients treated (2.2%), including one adjacent INF.

The cumulative incidence of local relapse at 5 years was 21.9% (95% CI 12.7-32.7), which is consistent with the rates found in literature, which encourages further dose escalation trials. The RTOG 0617 trial did not show any benefit from dose escalation at 74 Gy over the entire volume (36, 38). The current trend is to perform more selective dose escalation trials, particularly focusing on the most hypermetabolic tumor volume on the initial PET scan (41), the remaining hypermetabolic volume on the PET scan performed at mid-irradiation (42), or isotoxic radiotherapy, which consists of dose escalation until the constraints of the organ at risk are reached. In these trials, IMRT was chosen to limit the dose to organs at risk, particularly the heart, which seems to be a major predictive factor for survival (38, 43).

In the absence of tumor motion management, our treatment delivery may have involved increased dosimetric uncertainties.

We did not perform 4D CT when planning our treatment, so the tumour movement during the respiratory cycle was not modelled and we were unable to generate an internal target volume (ITV). However, in our centre, in order to limit irradiation volumes, we did not choose to generate an artificial ITV by adding a margin to the CTV. Only the margin added to the GTV according to the histological subtype and the 5 mm margin added to the CTV to generate the PTV were performed.Although the use of 4D CT with ITV would lead to a more appropriate treatment, the results in terms of local recurrence in our population remain comparable to Martinussen’s study.

Another theoretical approach to overcome the IMRT and tumor motion uncertainties (which include the blurring effect, interplay effect, and distortion of the dose distribution (31, 32)) is respiratory gating, which consists of controlling the patient’s breathing during treatment delivery. The prospective Gating 2006 study (44, 45) compared two groups of patients with NSCLC treated with radiochemotherapy with or without respiratory gating and found no differences in terms of OS, PFS, or toxicities. However, these patients were treated exclusively with 3D-CRT, and the results were not entirely extrapolatable to IMRT treatments.

The toxicities observed in the present study were acceptable. We counted five grade 3 radiation pneumonitis (7%), which is comparable with literature data with rates between 3.5% and 11% (16, 23, 46), and one grade 3 radiation esophagitis (1.5%), which is low compared with literature data with rates between 10.3% and 28% (16, 24, 47). However, these studies were based on the CTCAE 3.0 classification, which classifies grade 3 esophagitis as symptoms leading to calorically inadequate oral feeding. In our study, we classified grade 3 esophagitis as requiring enteral nutritional support, which was the only factor that could be retrospectively assessed when the esophagitis grade was not specified. We found 23% of grade 2 esophagitis, which we defined as the need for the introduction of a symptomatic treatment in connection with an important discomfort, and which could approach the grade 3 of the CTCAE 3.0 classification. We did not observe any immediate on-treatment cardiac toxicity or death related to cardiovascular pathology despite the prolonged median follow-up. However, the retrospective nature of the study does not guarantee exhaustive data.

These data remain monocentric and retrospective, with many biases that may artificially increase clinical outcomes and would need to be confirmed in a prospective randomized phase III trial. However, controlled clinical trials comparing IMRT and 3D-CRT in lung cancers would be difficult to perform because IMRT improves the benefit–risk ratio for patients. Moreover, conducting clinical trials comparing the two techniques would no longer be considered ethical. Therefore, IMRT has been implemented by most research teams and is currently widely used in lung cancer treatment. However, our results must be balanced by the small size of the study; therefore, it lacks statistical power.

In conclusion, this study suggests that IMRT is a safe technique for the treatment of locally advanced NSCLC with radiochemotherapy, with encouraging OS results, despite the absence of adjuvant immunotherapy at the time of the study. We did not find any significant excess risk of INF, which indicates that selective irradiation is a safe technique, regardless of lower mediastinal incidental irradiation. Toxicities remained acceptable. Despite many uncertainties regarding the optimal irradiation technique, clinical research has focused on individualized dose escalation (43, 44), fractionation, stereotactic boost of the primary tumor, and its association with innovative therapies. Given the promising results of consolidation immunotherapy with durvalumab, numerous studies have evaluated the combination and best therapeutic sequence of radiochemotherapy with immunotherapy, targeted therapies, or even bi-immunotherapy. Therefore, the management of locally advanced NSCLC is expected to evolve in the future.

## Data availability statement

The raw data supporting the conclusions of this article will be made available by the authors, without undue reservation.

## Ethics statement

The studies involving humans were approved by The study complies with the “reference methodology” MR004 adopted by the French Data Protection Authority. The patients granted consent to the use of their clinical data for research purposes. The study was approved by an international review board (CEC-2022-004). The studies were conducted in accordance with the local legislation and institutional requirements. The participants provided their written informed consent to participate in this study.

## Author contributions

TLe designed the work and wrote the manuscript. JW, MB and CL analyzed and interpreted the patient data. TLa, DP and EL was responsible for the management and coordination of the planning and execution of research activities. FL and MB revised the manuscript. All authors contributed to the article and approved the submitted version.
