# Supplementary material for: IMRT in the treatment of locally advanced or inoperable NSCLC in the pre-durvalumab era: clinical outcomes and pattern of relapses, experience from the Oscar Lambret Center
Source: Front Oncol. 2023 Sep 20;13:1236361. doi: 10.3389/fonc.2023.1236361 (PMC10554937; doi:10.3389/fonc.2023.1236361)
Supplement: Supplementary file 1 [file Table_1.docx]

Supplementary Material

IMRT in the treatment of locally advanced NSCLC: clinical outcomes and pattern of relapses, experience from the Oscar Lambret Center

Thomas Le Roy, Jennifer Wallet, Maël Barthoulot, Clémence Leguillette, Thomas Lacornerie, David Pasquier1, Eric Lartigau1, Florence Le Tinier*

*** Correspondence:** Dr. Florence Le Tinier, Academic Department of Radiation Oncology, Centre Oscar Lambret, Lille University, 3 Rue Combemale, 59020 Lille Cedex, France
Email: [f-letinier@o-lambret.fr](mailto:f-letinier@o-lambret.fr)

# Supplementary Table

Table S1. Early and late toxicities reported

| Toxicities reported | n |
| --- | --- |
| Early toxicities |  |
| Radiation pneumonitis | 35 |
| Grade 1 | 29 |
| Grade 2 | 1 |
| Grade 3 | 5 |
| Grade 4-5 | 0 |
| Radiation esophagitis | 43 |
| Grade 1 | 26 |
| Grade 2 | 16 |
| Grade 3 | 1 |
| Grade 4-5 | 0 |
| Pericarditis | 0 |
| Late toxicities |  |
| Esophageal stricture | 4 |
| Grade 1 | 0 |
| Grade 2 | 3 |
| Grade 3 | 1 |
| Radiation plexopathy | 1 |
| Grade 1 | 0 |
| Grade 2 | 1 |
| Oesobronchial fistula | 2 |
| Cardiovascular event | 0 |
